# 3-Gene-TB-SCORE Accuracy for Tuberculosis Disease Diagnosis Is Not Affected by Immune-Mediated Inflammatory Disease Comorbidity

**DOI:** 10.3390/ijms262210931

**Published:** 2025-11-12

**Authors:** Elisa Petruccioli, Tonino Alonzi, Assunta Navarra, Anna Maria Gerarda Altera, Gilda Cuzzi, Chiara Farroni, Federica Repele, Gina Gualano, Cecilia S. Lindestam Arlehamn, Fabrizio Palmieri, Andrea Salmi, Valentina Vanini, Delia Goletti

**Affiliations:** 1 Translational Research Unit, National Institute for Infectious Diseases Lazzaro Spallanzani-IRCCS, 00149 Rome, Italydelia.goletti@inmi.it (D.G.); 2 Epidemiology Unit, National Institute for Infectious Diseases Lazzaro Spallanzani-IRCCS, 00149 Rome, Italy; 3 Respiratory Infectious Diseases Unit, National Institute for Infectious Diseases Lazzaro Spallanzani-IRCCS, 00149 Rome, Italy; 4 Center for Vaccine Innovation, La Jolla Institute for Immunology, La Jolla, CA 92037, USA; 5 Center for Vaccine Research, Department of Infectious Disease Immunology, Statens Serum Institut, DK-2300 Copenhagen, Denmark; 6 Unità Operativa Semplice (UOS) Professioni Sanitarie Tecniche, National Institute for Infectious Diseases Lazzaro Spallanzani-IRCCS, 00149 Rome, Italy

**Keywords:** tuberculosis, RNA, signatures, biomarkers, TB-SCORE, TB, diagnosis

## Abstract

Tuberculosis (TB), caused by *Mycobacterium tuberculosis* (Mtb), remains a major global health threat. Approximately one-quarter of the world’s population has an Mtb-specific immune response and are classified as having tuberculosis infection (TBI), with a lifelong 5–10% risk of developing TB disease. This risk is elevated in individuals with immune-mediated inflammatory diseases (IMID) undergoing immunosuppressive therapies. To evaluate the diagnostic accuracy of the 3-gene TB-SCORE for TB disease in individuals within the TB spectrum, including those with TBI-IMID in a low TB endemic country, we prospectively enrolled 104 individuals with TB, TBI, TBI-IMID, and healthy donors. The 3-gene TB-SCORE and Mtb-specific response were evaluated and correlated with the participant’s clinical status. Patients with TB disease showed a significantly lower TB-SCORE compared to other cohorts. ROC analysis showed moderate diagnostic accuracy for TB disease (AUC 0.70–0.71). TB-SCORE was unaffected by IMID status or timing of Mtb exposure. Mtb-specific CD4^+^ T cells had no correlation to TB-SCORE. This is the first evaluation of TB-SCORE as a diagnostic tool for TB disease in a low-endemic setting. While further validation is needed, our findings support its potential as a biomarker for TB disease, even in complex clinical settings involving IMID.

## 1. Introduction

*Mycobacterium tuberculosis* (Mtb), the etiological agent of tuberculosis (TB), was estimated to be responsible for over 10 million TB cases and 1.25 million deaths in 2023 [1]. It is estimated that, globally, one-quarter of the population has an Mtb-specific immune response and are classified as presenting with tuberculosis infection (TBI), defined as a state of infection without clinical, microbiological, or radiological evidence of TB disease [2]. Individuals with TBI have a lifelong 5–10% risk of the reactivation of Mtb replication, leading to TB disease [1,3].

Patients with immune-mediated inflammatory diseases (IMID), such as rheumatoid arthritis (RA), may have increased susceptibility to infections, including TB, due to underlying immune dysregulation.

Among TBI-IMID patients, those with RA who are not on immunomodulatory or immunosuppressive therapy have a TB risk 2.0–8.9 times higher than TBI patients without IMID. In comparison, individuals with PsA or AS show a lower TB risk [4].

On the other hand, TB preventive therapy is mandatory for TBI-IMID patients undergoing treatment with biologic disease-modifying antirheumatic drugs (bDMARDs) or targeted synthetic DMARDs (tsDMARDs), including TNF-α inhibitors, anti-IL-6 agents, and JAK inhibitors [5,6,7]. Both bDMARDs and tsDMARDs may increase the risk of TB reactivation, although a fourfold increased risk has been specifically associated only with anti-TNF-α therapies [5]. Therefore, before initiating immunosuppressive treatment, RA patients are screened for TBI using the tuberculin skin test (TST) and/or interferon-γ release assays (IGRAs). If either test is positive, a chest X-ray is performed to exclude TB disease; if TBI is confirmed, TB preventive therapy is recommended [4,8,9,10].

To reduce global TB incidence, the World Health Organization (WHO) has proposed several strategies, including the development of non-sputum-based diagnostic tools for the triage, diagnosis, and prediction of TB progression [8,11]. Host transcriptomic tests have emerged as a potentially useful tool that, at least in part, may satisfy the accuracy criteria within the WHO target product profiles (TPPs) for TB diagnosis [12].

Despite the advantages of non-sputum-based diagnostics, transcriptomic approaches face limitations, including variable performance, high cost, technical complexity, and the heterogeneity of Mtb infection and host immune responses [12].

At present, none of the blood-derived gene expression signatures identified in recent years satisfy the minimum TPP criteria for predicting incident TB up to two years prior to clinical diagnosis [11,13,14,15,16,17,18]. Nevertheless, several RNA-based transcriptional signatures have met the minimal TPP thresholds for short-term prediction, specifically within a 3 to 12-month window preceding TB diagnosis [13,14,19,20].

A multicohort study conducted in Africa evaluated 20 published mRNA signatures for use as non-sputum TB triage tests. While none met the TPP criteria for TB disease diagnosis in pooled analyses, several met the minimum criteria in specific countries, underscoring the challenge of achieving reproducibility across diverse populations [21].

To address this, Cepheid developed a prototype GeneXpert PCR-based whole blood test using the TB-score, a three-gene signature proposed by Sweeney et al. in 2016 [22]. This assay, known as the Xpert MTB Host Response cartridge (not yet commercialized), measures the expression of three genes: guanylate binding protein 5 (*GBP5*), dual specificity phosphatase 3 (*DUSP3*), and Kruppel-like factor 2 (*KLF2*). This gene set is commonly referred to as the RISK-3 signature. *GBP5* and *DUSP3* are involved in pro-inflammatory responses and are upregulated during TB disease [23]. *GBP5* also mediates interferon responses in viral infections such as influenza A and HIV-1 [24,25], while *DUSP3* regulates innate immunity via the ERK1/2 pathway, TNF production, and macrophage polarization [26]. In contrast, *KLF2* acts as a negative regulator of monocyte activation [27].

A study conducted in HIV-infected individuals in South Africa and Peru demonstrated that the Xpert MTB-HR prototype performed effectively as a triage test using stored PAXgene blood samples [28]. However, a retrospective study in Brazil did not meet the TPP criteria for a triage test [29]. More recently, Sutherland and colleagues showed that the Xpert MTB Host Response assay met the minimum TPP requirements for a point-of-care TB triage test, regardless of participants’ geographic origin or HIV status [23]. Notably, no studies on the TB-SCORE are currently available from low TB-endemic countries.

In recent years, numerous studies have investigated Mtb-specific immune response as a tool to diagnose different TB statuses. Polyfunctional CD4^+^ T cells capable of simultaneously producing pro-inflammatory cytokines have been extensively examined as potential correlates of protection [30,31,32,33,34]. Due to the inconclusive nature of these findings, attention has shifted toward other aspects of the immune response, including the memory phenotype and activation status of antigen-specific T cells [35,36,37,38,39,40,41].

In this study, we aim to evaluate, in a low TB-endemic country, the TB-score as an assay to support TB diagnosis in a cohort of subjects with TB, TBI, or healthy donors. Given the elevated risk of TB progression in individuals with TBI and IMID (TBI-IMID), we also evaluate the TB-score in this vulnerable population. Additionally, in a subset of individuals, we characterized the Mtb-specific CD4^+^ T cells to explore their potential correlations with TB-score levels. Our findings confirm that patients with TB disease have a significantly lower TB-score compared to individuals with TBI and HD, even in a low TB-endemic setting such as Italy.

Furthermore, we demonstrated that the presence of IMID does not significantly affect TB-score values in individuals with TBI. While TB-score does not yet meet the WHO TPP criteria for triage testing for TB disease [8,11], its reliable performance across various cohorts underscores its promise as a supplementary tool for TB disease diagnosis in TB screening protocols.

## 2. Results

### 2.1. Characteristics of the Population

A total of 104 individuals within the TB spectrum, with or without IMID, as well as healthy donors (HD), were prospectively enrolled in the study (Table 1). A significant age difference was observed among the groups (*p* = 0.0014). Approximately 56% of participants were from Western Europe, and 43% had received BCG vaccination. Among patients with TBI-IMID, the majority (48%) had rheumatoid arthritis (RA), and 76% were receiving IMID therapy at the time of enrolment.

### 2.2. Patients with TB Disease Have a Significantly Lower TB-SCORE Compared to TBI and HD Individuals

**Figure 1:**
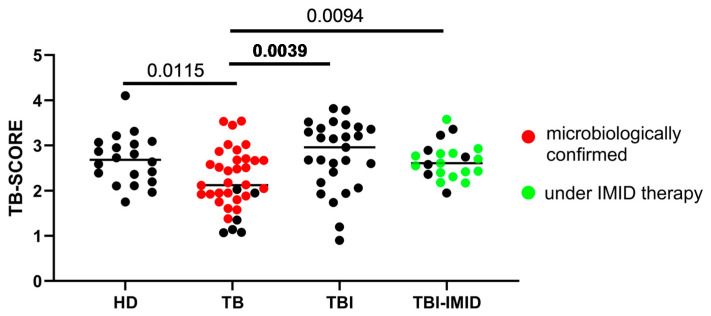
A low TB-SCORE is associated with patients with tuberculosis disease (TB). The TB-SCORE was calculated using the formula: (Ct GBP5 + Ct DUSP3)/2 − Ct KLF2. Healthy donors, patients with TB, and individuals with TBI, with and without IMID were evaluated. Significant difference after Bonferroni correction was reported in bold (*p* ≤ 0.008 for comparisons involving 4 groups). Abbreviations: HD: healthy donors, TB: tuberculosis disease, TBI: individuals with tuberculosis infection, IMID: immune-mediated inflammatory diseases.

The accuracy of the 3-gene TB-SCORE for identifying TB disease was evaluated in three prospective cohorts that included individuals with varying TB statuses and healthy donors (HD). Patients with TB disease had a significantly lower TB-SCORE compared to individuals with TBI, both with and without IMID (*p* = 0.0039 and *p* = 0.0094, respectively), as well as compared to HD (*p* = 0.0115) (Figure 1). Notably, no significant differences were observed between the TBI, TBI-IMID, and HD groups.

Quantile regression analysis (Table 2) corroborated these results, demonstrating a statistically significant positive difference in the estimated median TB-SCORE values for all groups compared to the reference category of TB patients. Furthermore, the findings were substantiated by the lack of significant differences in estimated median TB-SCORE values with respect to age, sex, and origin.

As 74% of TBI-IMID patients were receiving IMID therapy at the time of enrolment, we assessed the TB-SCORE after excluding those not undergoing IMID therapy. No statistically significant differences emerged between TBI-IMID patients on IMID therapy and TBI individuals. However, the TB-SCORE in this TBI-IMID group remained significantly higher compared to patients with TB disease (*p* = 0.0496; Supplementary Figure S1). Quantile regression analysis indicated that there were no significant differences in the estimated median TB-SCORE values between TBI-IMID patients who received IMID therapy and those who did not (see Supplementary Table S1).

Moreover, we conducted an in-depth analysis of the TB cohort, stratifying the results according to the clinical characteristics of the patients. TB patients with a clinical diagnosis exhibited significantly lower TB-SCORE values (*p* = 0.0013) compared to those with microbiologically confirmed TB (Supplementary Figure S2A). These results were confirmed by quantile regression analysis (Supplementary Table S2), which showed a significant reduction of 1.09 points (95% CI: −1.82; −0.37) in the median estimated TB-SCORE among patients with a clinical diagnosis. Furthermore, when assessing the number of positive TB-SCORE results, we found that all patients with a negative TB-SCORE had a confirmed microbiological diagnosis (*p* = 0.024; Supplementary Table S3).

In contrast, patients identified as having mild versus moderate to high TB severity demonstrated comparable TB-SCOREs (Supplementary Figure S2B and Supplementary Table S2), and no significant differences were detected in the number of positive TB-SCORE results across severity grades (Supplementary Table S3).

### 2.3. IMID Status Does Not Influence TB-SCORE Assessment Within the TBI Cohort

**Figure 2:**
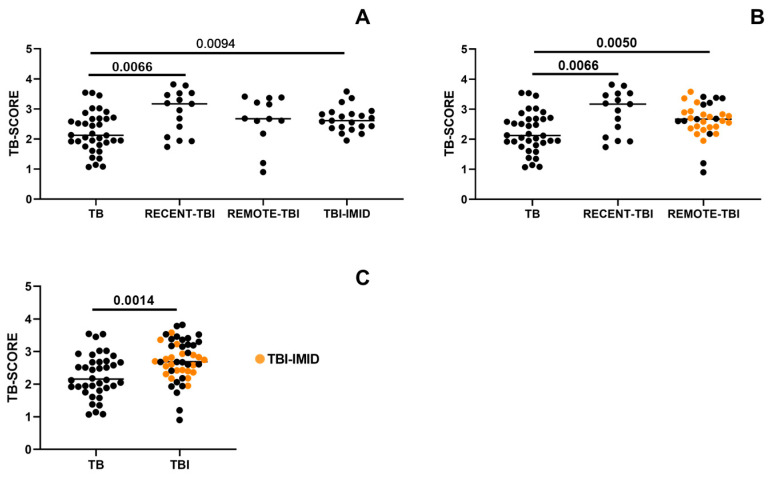
TBI patients exhibit a higher TB-SCORE compared to TB patients, regardless of IMID status or the timing of Mtb exposure. The TB-SCORE was calculated using the following formula: (Ct GBP5 + Ct DUSP3)/2 − Ct KLF2. Patients with TB, and individuals with TBI, with and without IMID were evaluated. (**A**) TBI individuals were stratified according to the timing of Mtb-exposure into RECENT-TBI and REMOTE-TBI. (**B**) TBI-IMID individuals having a remote Mtb exposure were included in REMOTE-TBI group. (**C**) TBI cases with remote exposure, with or without IMID, were pooled with TBI with recent exposure. Significant difference after Bonferroni correction is reported in bold (*p*-value ≤ 0.008 for comparisons involving 4 groups; *p* ≤ 0.016 for comparisons involving 3 groups; *p* ≤ 0.05 for comparisons involving 2 groups). Abbreviations: TB: tuberculosis disease, TBI: individuals with tuberculosis infection, IMID: immune-mediated inflammatory diseases.

We investigated whether the timing of acquisition of TBI could influence the TB-SCORE. When stratifying individuals with TBI based on the estimated duration from Mtb exposure, TB patients exhibited significantly lower TB-SCORE only when compared to TBI patients with recent exposure (*p* = 0.0066; Figure 2A). However, since TBI-IMID individuals, who had remote Mtb exposure, had a TB-SCORE comparable to both recent and remote TBI groups, they were included in the REMOTE-TBI category. This combined group exhibited a significantly higher TB-SCORE than patients with TB disease (*p* = 0.0050; Figure 2B).

Since neither the timing of Mtb exposure nor the presence of IMID significantly impacted TB-SCORE values, individuals with TBI and TBI-IMID were combined into a single cohort. This analysis confirmed that TBI individuals exhibited a significantly higher TB-SCORE than TB patients (*p* = 0.0014; Figure 2C).

### 2.4. Accuracy of TB-SCORE to Detect TB Cases

**Figure 3:**
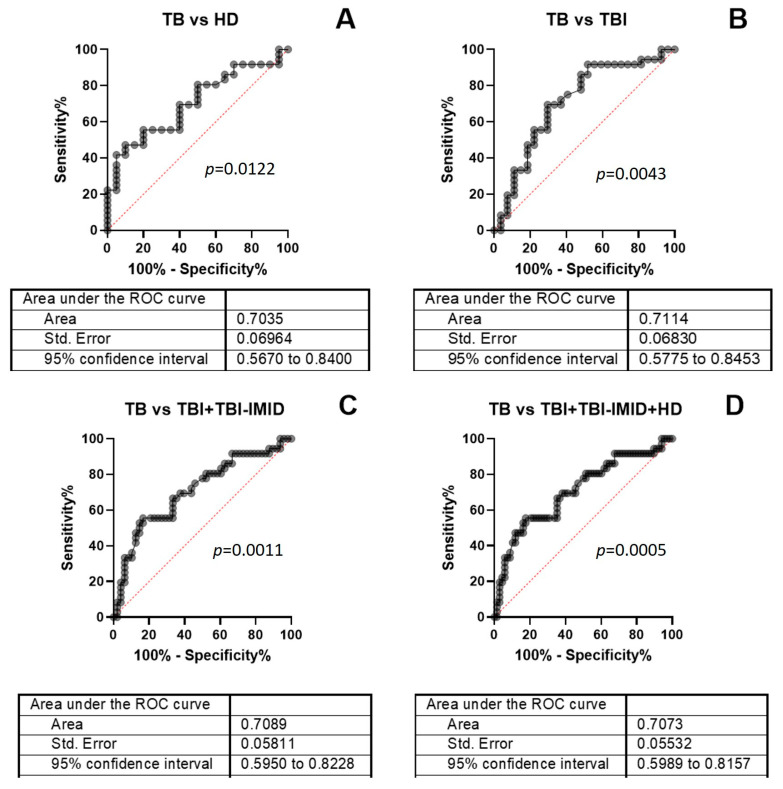
ROC analysis of TB-SCORE results. ROC curves were generated by comparing TB patients to (**A**) HD, (**B**) TBI, (**C**) the combined TBI + TBI-IMID group, and (**D**) the combined TBI + TBI-IMID + HD group. Optimal cut-off values were determined for each comparison to ensure a minimum specificity of 70%. Abbreviations: TB: tuberculosis disease, TBI: individuals with tuberculosis infection, IMID: immune-mediated inflammatory diseases, ROC: Receiver Operating Characteristic, AUC: area under the curve.

To evaluate the diagnostic accuracy of the TB-SCORE, we performed a receiver operating characteristic (ROC) curve analysis comparing TB patients to HD (AUC = 0.7035, 95% CI: 0.567–0.840, *p* = 0.0122), TB patients to individuals with TBI (AUC = 0.7114, 95% CI: 0.577–0.845, *p* = 0.0043), TB patients to the combined TBI + TBI-IMID groups (AUC = 0.7089, 95% CI: 0.595–0.823, *p* = 0.0011), and TB patients to the combined TBI + TBI-IMID + HD groups (AUC = 0.7073, 95% CI: 0.599–0.816, *p* = 0.0005) (Figure 3). Threshold values for each comparison were determined by maximizing sensitivity, with a requirement that specificity remained at or above 70% (Figure 3, Supplementary Table S4). Although the TB-SCORE did not meet the TPP criteria for a triage TB test as defined by the WHO (i.e., 90% sensitivity and 70% specificity), our findings indicate that the presence of IMID has minimal impact on the test’s diagnostic accuracy.

### 2.5. Antigen-Specific Immune Response of Individuals with TB Disease, TBI, and TBI-IMID

**Figure 4:**
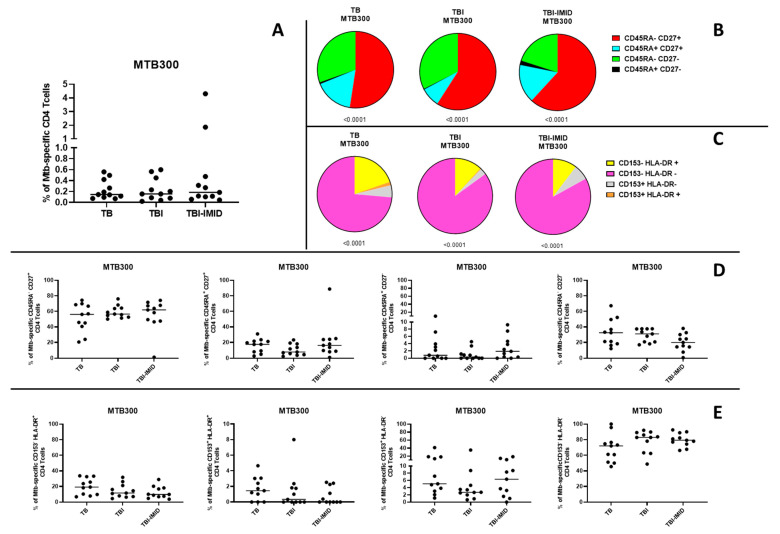
Evaluation of Mtb-specific CD4 T-cell responses in TB and TBI subjects with and without IMID. Peripheral blood mononuclear cells (PBMCs) were stimulated with Mtb-specific antigens (MTB300) for 24 h, and the immune response was assessed by flow cytometry. All analyses were conducted only among responders. (**A**) Antigen-specific responses were evaluated based on CD4 T cells producing IFN-γ, TNF-α, and/or IL-2. (**B**) Pie charts show the distribution of CD45RA and CD27 expression on Mtb-specific CD4 T cells. (**C**) Pie charts show the distribution of CD153 and HLA-DR expression on Mtb-specific CD4 T cells. (**D**) Graphs compare the frequency of Mtb-specific CD4 T cells expressing CD45RA and/or CD27 across groups. (**E**) Graphs compare the frequency of Mtb-specific CD4 T cells expressing CD153 and/or HLA-DR across groups. The Wilcoxon matched-pairs signed-rank test and the Kruskal–Wallis test were used for matched and unmatched comparisons, respectively. Abbreviations: TB: tuberculosis; TBI: tuberculosis infection; IMID: immune-mediated inflammatory disease.

To assess the capacity of enrolled individuals to mount a Mtb-specific immune response, we analyzed CD4^+^ T cell responses to a peptide pool of Mtb-derived T cell epitopes (MTB300) in a subset of participants previously evaluated for the TB-SCORE (TB = 11; TBI = 11; TBI-IMID = 11). Mtb-specific CD4^+^ T cells were identified based on the production of IFN-γ, TNF-α, and/or IL-2 (Figure 4A and Supplementary Figure S3). In addition, we analyzed the expression of CD45RA, CD27, CD153, and HLA-DR within the Mtb-specific CD4^+^ T-cell population to characterize the memory and activation status of these cells (Figure 4B–E; Supplementary Figure S3). All groups demonstrated the ability to respond to Mtb-specific stimulation (Figure 4A), with the predominant phenotype CD45RA^−^ CD27^+^ (Figure 4B,D) or CD153^-^ HLA-DR^−^ (Figure 4C,E) among Mtb-specific CD4^+^ T cells. No significant differences were observed in the distribution of CD45RA^−/+^ CD27^−/+^ CD153^−/+^ HLA-DR^−/+^ Mtb-specific CD4^+^ T-cell subsets across the groups (Figure 4D,E).

We did not find any significant correlation between the frequency of Mtb-specific CD4^+^ T cells and the TB-SCORE (Supplementary Figure S4).

## 3. Discussion

In this study, we conducted, in a low TB endemic country, a prospective assessment of the accuracy of the 3-gene TB-SCORE for diagnosing TB disease in a cohort encompassing individuals across the TB spectrum, including those with IMID and HD. Our findings confirm that patients with TB disease have a significantly lower TB-SCORE compared to individuals with TBI as well as to HD. These results are consistent with previous studies performed in high TB endemic countries, demonstrating the discriminatory potential of host transcriptomic signatures in identifying TB disease [20,23,28,42,43].

The analysis of the clinical characteristics of TB patients indicates that a microbiological diagnosis is significantly associated with a higher TB-SCORE compared with the clinical diagnosis. Assuming that a microbiological diagnosis reflects a high Mtb load, these results correlate with the extent of lung involvement. A previous study reported elevated TB-SCOREs in patients with persistent lung inflammation at the end of treatment [20]. However, in our study, the analysis of TB-SCORE in relation to TB severity did not show significant differences between groups.

Importantly, we observed that the IMID comorbidity did not significantly affect TB-SCORE values of TBI with respect to those of TB patients, nor did the estimated timing of Mtb exposure (i.e., remote vs. recent). This allowed us to group TBI and TBI-IMID individuals, reinforcing the robustness of the TB-SCORE in identifying TB patients, regardless of the immunological state of the diverse populations. These findings are particularly relevant for TBI-IMID, given the increased risk of TB reactivation in patients undergoing immunosuppressive therapy, especially those treated with TNF-α inhibitors [5].

The diagnostic accuracy for TB disease of the TB-SCORE was further assessed through ROC curve analysis, achieving moderate discriminatory power (AUCs ranging from 0.70 to 0.71). The accuracy was consistent across comparisons, and the IMID comorbidity did not compromise the test’s accuracy, suggesting its potential utility in clinical settings where IMID patients are routinely screened for TBI before immunosuppressive treatment.

A key strength of this study is that it constitutes the first evaluation of the TB-SCORE in a low TB-endemic country. Previously, most research has focused on high-burden settings, where both underlying immune activation and TB exposure are more common. Our results indicate that the TB-SCORE maintains its discriminatory ability for diagnosing TB disease versus either TBI or HD, even in a low-incidence environment.

However, while the TB-SCORE does not yet meet the WHO Target Product Profiles (TPPs) criteria (90% sensitivity and 70% specificity) for a triage TB test [8], it consistently identifies TB disease across diverse cohorts. When the cut-off was selected to ensure a specificity of at least 70%, thus meeting one of the TPP’s minimum requirements, the TB-SCORE reached a sensitivity of 56% and a specificity of 80%. Although this reflects a reduced accuracy in low TB prevalence settings, the TB-SCORE’s consistent performance underscores its potential value as a complementary tool within TB screening strategies.

Our functional analysis of Mtb-specific CD4^+^ T-cell responses confirmed that all groups retained the capacity to mount an immune response to Mtb antigens, with a predominant CD45RA^−^ CD27^+^ and CD153^−^ HLA-DR^−^ phenotype. However, no clear association was observed between the intensity of the Mtb-specific immune response and the TB-SCORE, which may suggest that the transcriptomic profile reflects broader immune system activation rather than specific antigen-driven responses. This interpretation, however, requires further validation in larger study populations.

While promising, transcriptomic approaches still face challenges related to cost, technical complexity, and variability across populations [12,21]. These factors are particularly critical when considering their integration into everyday clinical workflows, especially in resource-limited settings. Our study did not explore the kit availability or logistical feasibility of implementing the TB-SCORE in such environments—an aspect that is crucial for its potential adoption in public health programs. Future investigations should therefore incorporate cost–benefit analyses and operational assessments to determine whether the TB-SCORE can be realistically and sustainably applied in routine care, particularly where infrastructure and funding are constrained. However, our findings support the continued development and validation of simplified, point-of-care-compatible assays such as the Xpert MTB-HR, which has shown encouraging results in recent multicenter evaluations [23].

Another important point raised by our work is the demonstration that the presence of IMID does not significantly influence TB-SCORE values, at least among individuals with TBI. This finding has important implications for future clinical trials and diagnostic validation studies. Specifically, it suggests that the inclusion of IMID patients within TBI cohorts is unlikely to introduce bias or confounding in the evaluation of host-response biomarkers such as the TB-SCORE. As a result, future studies aiming to validate transcriptomic signatures for TB diagnosis or triage may confidently include IMID populations without compromising the interpretability or generalizability of their findings.

The observation that TB-SCORE values remain unaffected by IMID status provides valuable insight for longitudinal studies aiming to evaluate TB-SCORE as a biomarker for monitoring the efficacy of TB treatment [44,45]. This is an important point for future research that was not within the scope of our study, which did not include follow-up assessments.

This finding supports the potential utility of TB-SCORE in heterogeneous patient populations, including those with immune-mediated inflammatory diseases, where immune modulation could otherwise confound biomarker interpretation.

On the other hand, this study has some limitations. First, the relatively small sample size may limit the statistical power and generalizability of our findings. Larger, multicenter studies are needed to validate these results across broader and more diverse populations. Second, we employed a custom, research-based qPCR method to assess the TB-SCORE, whereas other studies have used standardized, cartridge-based platforms such as the Xpert MTB Host Response assay [23,28,42,43]. This lack of methodological standardization may affect the comparability of our results with those obtained using standardized diagnostic tools. Third, our analyses were conducted using frozen PBMCs rather than whole blood [20,23,28,42,43], which is the preferred sample type for transcriptomic assays intended for clinical application. The use of PBMCs may introduce variability due to cell isolation and processing steps and may not fully reflect the performance of the TB-SCORE in whole blood-based platforms designed for point-of-care use. Additionally, recent studies have shown that using bio-banked whole blood samples may result in lower median TB-SCORE values compared to fresh samples [46].

Our cohort included individuals from various geographic origins, with the exception of healthy donors, who were all from Western Europe. However, the quantile regression analysis demonstrated that geographic origin had no significant impact on TB-SCORE values. Nonetheless, expanding the sample size to include individuals from a broader range of geographic backgrounds could add substantial value to these findings and help overcome one of the main limitations of this study.

Despite the limitations related to sample size, the use of PBMCs instead of whole blood, and the application of a non-standardized qPCR method, our study provides valuable evidence supporting the utility of the 3-gene TB-SCORE in identifying TB disease. Notably, we demonstrate that the presence of IMID does not significantly affect TB-SCORE accuracy, reinforcing its potential applicability in immunocompromised populations.

These findings contribute to the growing body of evidence supporting host transcriptomic signatures as promising tools for TB disease triage and diagnosis. Although further validation with standardized, point-of-care platforms and larger cohorts is needed, our results suggest that TB-SCORE may serve as a biomarker for TB in low-endemic countries, even in complex cases involving IMID patients. Although it does not yet meet the WHO TPP criteria for triage testing, its consistent accuracy in identifying TB disease across diverse cohorts underscores its potential utility as a complementary component in TB screening strategies.

## 4. Materials and Methods

### 4.1. Study Population

This study was approved by the Ethical Committee of the INMI Lazzaro Spallanzani-IRCCS (approval numbers 72/2015 and 27/2019). Written informed consent was obtained from all participants.

In accordance with current guidelines [5], TBI screening in IMID patients was conducted prior to initiation or modification of biologic therapy due to non-clinical benefit. In the absence of clinical, microbiological, or radiological signs of TB disease, TBI was diagnosed based on a positive QuantiFERON (QFT)-Plus assay result (Diasorin, Vercelli, Italy).

TBI (*n* = 27) and TBI-IMID (*n* = 21) subjects were enrolled before starting TB preventive therapy. TB diagnosis was based on microbiological and radiological findings; TB patients (*n* = 36) were recruited before or within seven days of treatment initiation. Healthy donors (HD) with negative QFT-Plus results served as controls (*n* = 20). Table 1 summarizes demographic and clinical characteristics. The study adhered to the STROBE guidelines for case–control studies [47].

### 4.2. Chest X-Ray Evaluation

All chest X-rays, anonymized before analysis, were assessed for radiological signs including nodules, fibrosis, infiltrates, cavitation, bronchial dissemination, miliary patterns, pleural effusion, and lymphadenopathy, following established criteria. The diameter of cavities was recorded (<4 cm or ≥4 cm). The extent of lung involvement was estimated visually based on the proportion of parenchymal infiltrates, with a 30% threshold used internally to categorize disease severity. Based on published data and clinical experience, disease severity was graded by three independent reviewers (DG, FP, GG) using the following scale: Grade 0: normal chest X-ray; Grade 1: mild disease (nodules and/or infiltrates affecting <30% of the lung); Grade 2: moderate disease (infiltrates affecting >30% of the lung and/or cavities < 4 cm); Grade 3: severe disease (any extent of infiltrates with cavities ≥ 4 cm and/or presence of bronchial spread, miliary pattern, pleural effusion, or lymphadenopathy). All participants underwent standard chest radiography at the time of TB diagnosis.

### 4.3. PBMC Isolation and RNA Isolation

Peripheral blood samples (PBMC) were collected using heparinized BD Vacutainer^®^ tubes. PBMCs were isolated from HD, TB, TBI and TBI-IMID subjects using Ficoll gradient (Cedarlane Labs, Burlington, ON, Canada; Cat. No. CL5020-RC) with SepMate™ tubes (StemCell, Vancouver, BC, Canada; Cat. 85460), cryopreserved in heat-inactivated fetal bovine serum (FBS) supplemented with 10% DMSO and stored in liquid nitrogen. RNA was isolated from 1.0 to 3.0 × 10^6^ cells by RNeasy Kits (Qiagen, Hilden, Germany; Cat. No. 74106).

### 4.4. TB-Score Generation

Quantitative Reverse Transcription Polymerase Chain Reaction PCR (RT-PCR) reactions were performed using the KAPA SYBR FAST 1STEP UNI (Kapa Biosystems, Inc., Wilmington, MA, USA, Cat. No. KK4652) coupled to RotorGene 6000 real-time PCR system (Qiagen). Ct values for individual genes (*GBP5*, *DUSP3*, and *KLF2*) were used to calculate a TB score determined with the formula: [(Ct *GBP5* + Ct *DUSP3*)/2 − Ct *KLF2*] [22].

Primer sets for all amplicons were listed in Supplementary Table S5. An amount of 50 ng of RNA were used as template and cycling parameters were 42 °C for 5 min, 95 °C for 3 min, followed by 40 cycles of 95 °C for 3 s, 60 °C for 20s, and 72 °C for 30 s.

### 4.5. Stimulation and Reagents

Thawed PBMCs were cultured at a concentration of 0.5–1.0 × 10^6^ cells/mL in 96-well plates for 24 h at 37 °C, 5% CO_2_ in RPMI-1640 (Merck, Darmstadt, Germany, Cat. No. R0883), 10% FBS (Gibco, Life Technologies, Waltham, MA, USA, Cat. No. 10270106), 2 mM L-glutamine, and 1% penicillin/streptomycin. Cells were stimulated with a pool of 300 Mtb-derived peptides (MTB300, 1.5 µg/mL) [48] and co-stimulatory monoclonal antibodies α-CD28 and α-CD49 (1 µg/mL each; BD Biosciences, San Jose, CA, USA). BD Golgi Plug was added after one hour.

### 4.6. Intracellular Staining Assay and Flow Cytometry Analysis

Intracellular cytokine staining was performed after 24 h of incubation as previously described [35]: Fixable Viability Stain 700, CD3 V450 (clone UCHT1), CD8 APC-H7 (clone SK1), CD27 BV605 (clone L128), CD45RA PE-Cy7 (clone L48), HLA-DR BV786 (clone G46-6), IFN-γ APC (clone B27), IL-2 PerCP-Cy5.5 (clone MQ1-17H12), and TNF-α FITC (clone MAb11), (all from BD): CD4 ECD (Beckman Coulter, clone SFCI12T4D11), CD153 PE (R&D System, clone 116614). Brilliant Stain Buffer (BD) and Cytofix/Cytoperm (BD) were used according to the manufacturer’s instructions. At least 100,000 lymphocytes per sample were acquired using a DxFLEX flow cytometer (Beckman Coulter) and analyzed with FlowJo v10.8.1. Antigen-specific responses were deemed positive if stimulated cells were ≥2× the unstimulated control and ≥10 events were detected in the gated population. Analyses were performed blindly by two independent operators (EP and CF). Gating strategy is shown in Supplementary Figure S2.

### 4.7. Statistical Analysis

Data were analyzed using Graph Pad Prism (Version 8.2.1) and SPSS software (Version 29). The median and interquartile ranges (IQRs) were calculated for continuous measures. For pairwise comparison, Mann–Whitney U was used. Friedman’s test was used to compare paired data of antigen-specific response. Receiver Operator Characteristic (ROC) was used to determine the cut-off values and sensitivity/specificity of TB-SCORE results.

A quantile regression model for the median was used to evaluate differences in TB-SCORE values across different category (age, sex, origin, diagnosis, presence or not of IMID therapy, microbiological or clinical diagnosis and grade of severity).

The 95% confidence intervals were estimated using a non-parametric bootstrap method based on 2500 resamples. Regression analyses were performed using Stata (StataCorp., 2021. Stata: Release 17. Statistical Software. College Station, TX: StataCorp LLC, College Station, TX, USA). Quantile regression estimates the effect of covariates on the conditional median or other quantiles, providing a more comprehensive view of the relationship between variables, particularly in the presence of non-normal distributions or outliers, allowing us to adjust for confounding variables.

## Figures and Tables

**Table 1:** Clinical characteristics of the enrolled patients.

	HD N = 20	TB N = 36	TBI N = 27	TBI-IMID N = 21	TOTAL N = 104	*p* Value
**Age median (IQR)**	46 (39–53)	36 (28–49)	37 (23–54)	54 (48–66)	45 (31–54)	**0.0014** *
**Female N (%)**	12	17	13	14		0.443 ^#^
**Origin N (%)**						na
**West Europe**	20 (0)	11 (31)	13 (48)	14 (67)	58 (55.7)	
**East Europe**	0 (0)	13 (36)	9 (33)	4 (19)	26 (25)	
**Africa**	0 (0)	2 (5)	2 (8)	0 (0)	4 (4)	
**Asia**	0 (0)	6 (17)	3 (11)	1 (5)	10 (9.6)	
**South America**	0 (0)	4 (11)	0 (0)	2 (9)	6 (5.7)	
**BCG vaccination (%)**	0	24	14	7	45 (43)	na
**IMID N (%)**						
**Rheumatoid arthritis**				10 (48)		**0.0019 ^#^**
**Psoriatic arthritis**				8 (38)		
**Polymyalgia rheumatica**				1 (5)		
**Psoriasis**				2 (9)		
**under IMID therapy N (%)**				16 (76)		/
**IMID therapy N (%)**						
**B**				5 (31)		
**C**				3 (19)		na
**cDMARDs**				3 (19)		
**cDMARDs + C**				5 (31)		

Footnotes: N: Number; TBI: TB infection; TB: tuberculosis; IMID: inflammatory-mediated immune disease; IQR: interquartile range; B: Biological; C: Corticosteroids; cDMARDs: conventional DMARDs; BCG: bacillus Calmette et Guérin; na: not applicable, since Chi-square calculations are only valid when all expected values are greater than 1.0 and at least 20% of the expected values are greater than 5; * Kruskal- Wallis test; ^#^ Chi Square test; Significant *p* values are reported in bold.

**Table 2:** Quantile regression analysis reveals a significant positive median expected value of TB-SCORE in the HD, TBI, and TBI-IMID groups compared to TB patients.

		Coefficient (95% CI)	*p*
**Age**	(per 10-year increment)	−0.01 (−0.12; 0.11)	0.884
**Sex**	F vs. M	0.19 (−0.09; 0.46)	0.178
**Origin**	Eastern Europe vs. Western Europe	−0.25 (−0.75; 0.24)	0.308
	Asia vs. Western Europe vs. Western Europe	−0.09 (−0.83; 0.65)	0.810
	Africa vs. Western Europe	−0.49 (−1.49; 0.52)	0.338
	South America vs. Western Europe	−0.12 (−0.98; 0.75)	0.791
	All origins vs. Western Europe	−0.15 (−0.48; 0.18)	0.384
**Diagnosis**	HD vs. TB	0.60 (0.09; 1.10)	0.021
	TBI vs. TB	0.83 (0.21; 1.44)	0.009
	TBI-IMID vs. TB	0.48 (0.05; 0.91)	0.031
	TBI/TBI-IMID vs. TB	0.57 (0.14; 0.99)	0.010

Footnotes: TBI: TB infection; TB tuberculosis; IMID inflammatory-mediated immune disease, reference category: Male, Western Europe, TB. For the categorical variable the coefficient represents the difference in medians values of TB-score between the categories. For the continuous variable (age) the coefficient represents the variation in TB-score for 10 year increments of variable age.

## Data Availability

The datasets presented in this study can be found in online repositories. The raw data are available in our institutional repository (rawdata.inmi.it), subject to registration. The data can be found by selecting the article of interest from a list of articles ordered by year of publication. No charge for granting access to data is required. In the event of a malfunction of the application, the request can be sent directly by e-mail to the Library (biblioteca@inmi.it).

## Supplementary Materials

The following supporting information can be downloaded at: https://www.mdpi.com/article/10.3390/ijms262210931/s1.

## Abbreviations

The following abbreviations are used in this manuscript:

TB	Tuberculosis
TBI	Tuberculosis infection
IMID	immune-mediated inflammatory diseases
Mtb	*Mycobacterium tuberculosis*
