# Supplementary material for: 3-Gene-TB-SCORE Accuracy for Tuberculosis Disease Diagnosis Is Not Affected by Immune-Mediated Inflammatory Disease Comorbidity
Source: Int J Mol Sci. 2025 Nov 12;26(22):10931. doi: 10.3390/ijms262210931 (PMC12652831; doi:10.3390/ijms262210931)
Supplement: Supplementary file 1 [file ijms-26-10931-s001.zip › ijms-3883122-supplementary.pdf]

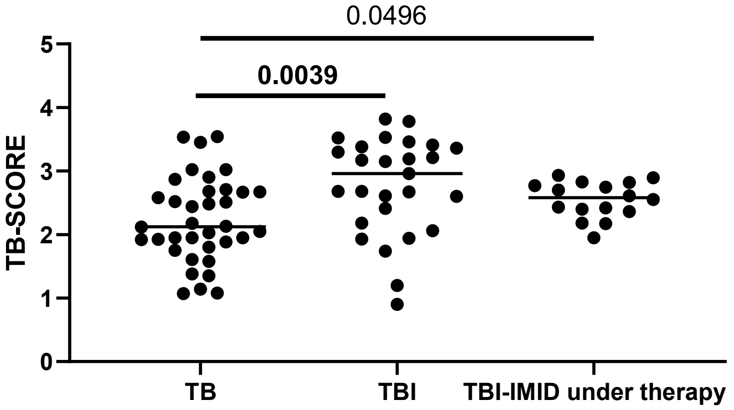

Supplementary Figure S1: TB-SCORE median of TBI-IMID patients receiving IMID therapy is similar to TBI individuals. The TB-SCORE was calculated using the formula: (Ct GBP5 + Ct DUSP3)/2 − Ct KLF2. Statistically significant differences after Bonferroni correction are reported in bold. Abbreviations: TB: tuberculosis disease, TBI: individuals with tuberculosis infection, IMID: immune-mediated inflammatory diseases

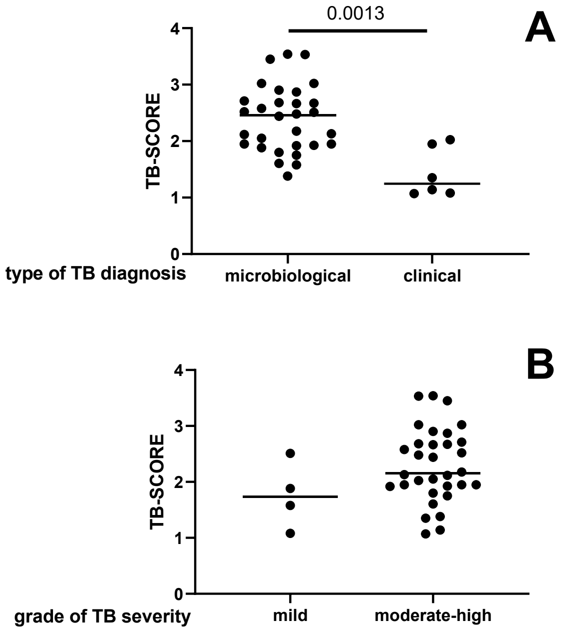

Supplementary Figure S2: TB-SCORE values in TB patients stratified according to clinical severity. A) microbiological diagnosis versus clinical diagnosis; B) TB severity grade. The TB-SCORE was calculated using the formula: (Ct GBP5 + Ct DUSP3)/2 − Ct KLF2. Statistically significant differences after Bonferroni correction are reported in bold. Abbreviations: TB: tuberculosis disease

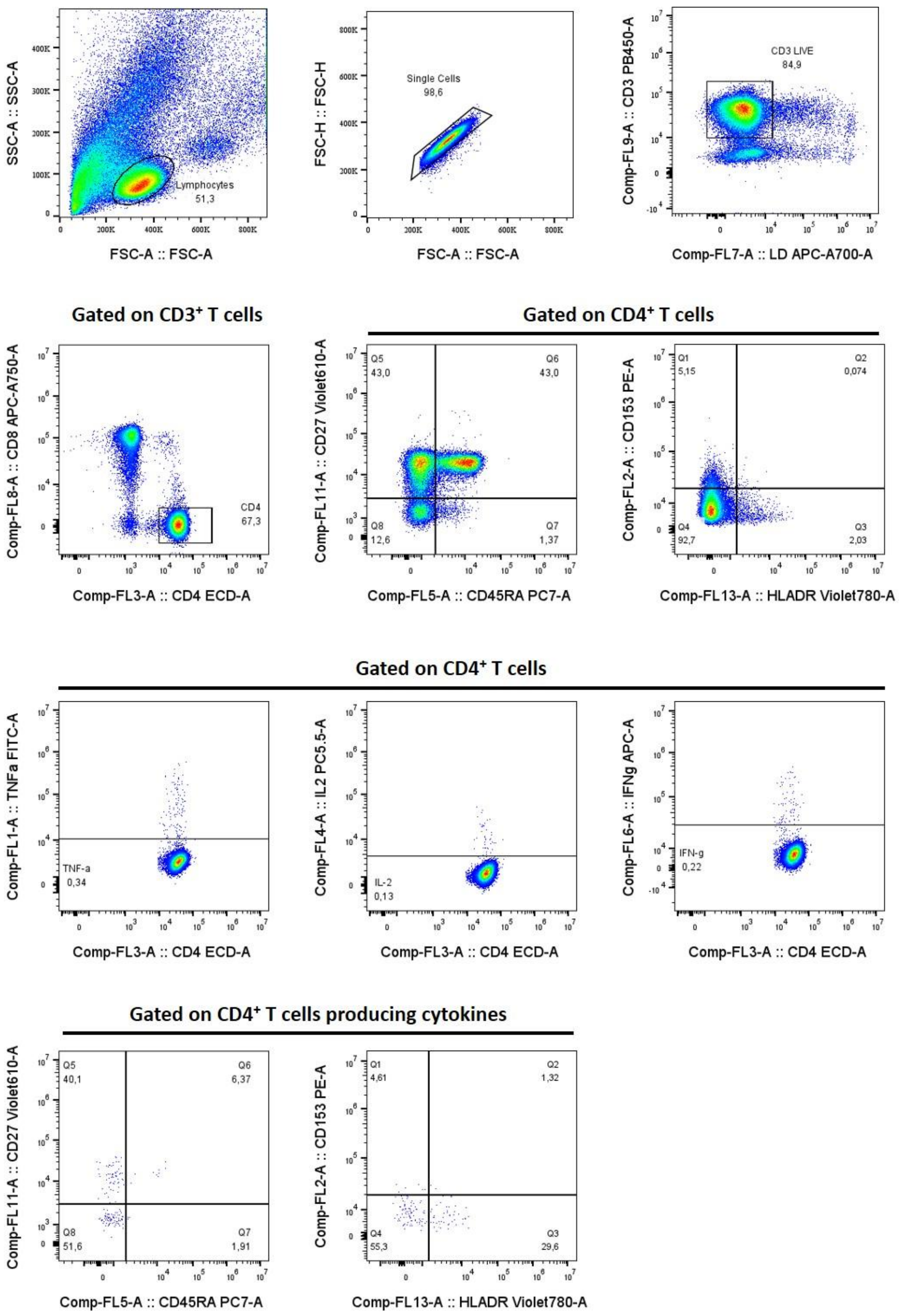

Supplementary Figure S3: Flow cytometry gating strategy for the characterization of memory and activation status of Mtb-specific CD4$^+$ T cells producing cytokines. A Boolean gate analysis was applied to evaluate CD4$^+$ T cells producing cytokines (IFN-γ and/or TNF-α and/or IL-2). The gating strategy shown is representative of a TB patient.

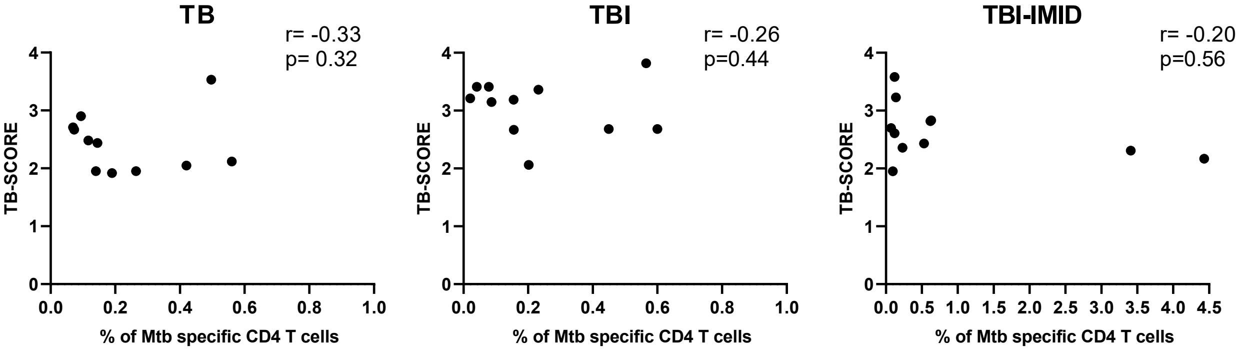

Supplementary Figure S4. Absence of correlation between TB-SCORE values and the magnitude of Mtb-specific CD4⁺ T-cells.

The relationship between the frequency of Mtb-specific $CD4^+$ T cells (x axis) and TB-SCORE values (y axis) was assessed across groups using Spearman's correlation.

Abbreviations: TB: tuberculosis; TBI: tuberculosis infection; IMID: immune-mediated inflammatory disease.

Supplementary Table S1. Quantile regression analysis reveals no difference in the median expected value of TB-SCORE in TBI-IMID patients under IMID therapy compared to patients not under therapy.

Comparison	Coefficient (95% CI)	p
cDMARDS vs no therapy	-0.87 (-1.86; 0.12)	0.081
Biological vs no therapy	-0.81 (-1.65; 0.04)	0.061
Corticosteroids vs no therapy	-0.48 (-1.31; 0.34)	0.230
Corticosteroids+cDMARDs vs no therapy	-0.53 (-1.49; 0.43)	0.256
Therapy vs no therapy	-0.68 (-1.51; 0.16)	0.106

Footnotes: TBI: TB infection; IMID: inflammatory mediated immune disease; cDMARDs: conventional DMARDs; CI: confidence interval; Reference category: no therapy;

Supplementary Table S2. Impact of clinical characteristic of TB patients on TB-SCORE values: quantile regression

Quantile regression**	Univariable	
	Coefficient (95% CI)	p
Clinical vs Microbiological diagnosis	-1.09 (-1.82; -0.37)	0.004
Low severity vs Moderate-to-high severity	-0.29 (-1.16; 0.57)	0.496

Footnotes: microbiological diagnosis and moderate to high severity were considered as reference category for quantile regression analysis, CI: confidence interval

Supplementary Table S3. Impact of clinical characteristic of TB patients on TB-SCORE results

Contingency table	Microbiological diagnosis	Clinical diagnosis	p §	Mild severity	Moderate-to-high severity	p§
Positive TB-SCORE*	14 (87,5)	6 (30)	**0.024**	5 (25)	15 (75)	0.200
Negative TB-SCORE*	16 (100)	0 (0)		1 (6)	15 (94)	

Footnotes: *TB-SCORE results were associated with TB diagnosis if < 2,187; §Fisher exact test, **microbiological diagnosis and middle high severity were considered as reference category for quantile regression analysis.

Supplementary Table S4. Diagnostic accuracy parameters of the TB-SCORE for TB disease, as determined by ROC analysis, including cut-off values, sensitivity, and specificity.

	Cut-off <	Sensitivity (95% CI)	Specificity (95% CI)
**TB vs HD**	2.187	55.6 (39.6-70.5)	80.0 (58.40-92.0)
**TB vs TBI**	2.590	69.4 (53.1-82.0)	70.4 (51.5-84.1)
**TB vs TBI+TBI-IMID**	2.179	55.6 (39.6-70.5)	83.3 (70.4-91.3)
**TB vs TBI+TBI-IMID+HD**	2.179	55.6 (39.6-70.5)	82.4 (71.6-90.0)

Footnotes: HD: healthy donors; TB: tuberculosis; TBI: TB infection; IMID: inflammatory mediated immune disease

Supplementary Table S5. Primer sets for all amplicons used in the study

Gene (Homo Sapiens)	Primers Sequence
GBP5	For 5’-AGCAGCTGAAGGTTAATCAGG-3’<br>Rev 5’-GGATTTGCCAGTGCGATAGA-3’
DUSP3	For 5’-CATCACATACCTGGGCATCA-3’<br>Rev 5’-AGCCAAAGCCTGGTCAAT-3’
KLF2	For 5’-GGCAAGACCTACACCAAGAG-3’<br>Rev 5’-GTCCCAGTTGCAGTGGTAG-3’

Footnotes: *GBP5: guanylate binding protein 5; DUSP3: dual specificity phosphatase 3 KLF2: Kruppel-like factor 2;* For: Forward; Rev: Reverse.
